# Bat Incidents at Children's Camps, New York State, 1998–2002

**DOI:** 10.3201/eid1102.040709

**Published:** 2005-02

**Authors:** Amy Robbins, Millicent Eidson, Mary Keegan, Douglas Sackett, Brian Laniewicz

**Affiliations:** * New York State Department of Health, Albany, New York, USA

**Keywords:** rabies, rabies vaccine, bats, chiroptera, public health surveillance, environmental exposure, dispatch

## Abstract

From 1998 to 2002, a total of 299 bat incidents were reported at 109 children's camps in New York; 1,429 campers and staff were involved, and 461 persons received rabies treatment. In 52.5% of the incidents, the bat was captured and samples tested negative for rabies virus, which resulted in 61.3% of persons not receiving rabies treatment.

Rabies is a neurologic disease with close to a 100% case-fatality rate; once clinical signs appear, it is almost always untreatable (*1*). After a person is exposed to rabies, death can be prevented only if treatment, commonly referred to as postexposure prophylaxis (PEP), is initiated. PEP includes an initial dose of immune globulin and a series of 5 doses of rabies vaccine in a 1-month period. PEPs are costly in terms of money and time because of the 5 medical visits, particularly if the person must be transported elsewhere for the treatment. The New York State Department of Health (NYSDOH) has a unique program that requires that rabies exposures and treatments be reported. County expenses associated with authorized treatments in accordance with state and federal guidelines are then partially reimbursed (*2*).

Despite a large number of rabid animals in the United States (7,967 confirmed in 2002), rabies in humans is rare because of the availability of PEP; 31 cases were reported in the United States from 1990 to 2003 (*3*). Twenty-nine (94%) of the 31 cases were associated with bat rabies variants, and a bat bite could be definitively documented for only 3 of them (*3*). Four children in the United States (*4**–**8*) and 1 child in Quebec, Canada, died of bat-related rabies (*9*). The families of the children in the United States were unaware of the potential for rabies transmission from bats.

Children's summer camps share habitats favored by bats and other wildlife; thus, children and camp staff may come into contact with bats that are either roosting in camp buildings or flying among camp facilities while foraging. A camp-related rabies death occurred in Alberta, Canada, in 1985 in a 25-year-old student who had been bitten and scratched by a bat and received no treatment (*10*).

Of the 3,827 bats tested by the NYSDOH Wadsworth Center's Rabies Laboratory in 2002, 102 (2.6%) were rabid (*11*). Although the probability of an individual bat being rabid is relatively low, bats that can expose humans to rabies must be assumed rabid, when a definitive diagnosis of rabies cannot be made. In 1999, the federal Advisory Committee on Immunization Practices (ACIP) updated the national PEP recommendations to include incidents with bats in which there was a "reasonable probability that exposure has occurred" (*12*). These types of incidents include direct contact with a bat; a bite, scratch, or mucous membrane contact with bat saliva or nervous tissue; a sleeping person awakening to find a bat in the room; or an adult witnessing a bat in the room with a previously unattended child, or a mentally disabled or intoxicated person (*12*).

## The Study

In 1998, the NYSDOH Zoonoses Program began an educational program to address the importance of bats in camp settings. This program was conducted in collaboration with the NYSDOH Center for Environmental Health (CEH), Bureau of Community Environmental Health and Food Protection (BCEHFP). NYSDOH offered training for all local and state health department camp inspectors responsible for inspecting camps before opening each season. Fact sheets on bats and bat-proofing camps and houses, bat capture kits, guidelines for managing bats, risk for rabies transmission (particularly in children's camp settings), and guidance regarding human exposure to rabies and treatment decisions were provided. Starting in 1999, these materials included rabies awareness refrigerator magnets instructing people to contact health departments and not release bats when they are found in dwellings, and rabies awareness stickers for children to teach them not to touch bats (*13*). In 2003, ≈700 children's camps received a videotape about keeping bats out of occupied dwellings and capturing bats for testing in exposure incidents.

Children's camp operators are required by New York State Public Health Law to obtain a permit, and camps must undergo inspection by the local health department. Associated regulations require camp operators to report certain camper injuries and illnesses within 24 hours of occurrence. Beginning in 1998, bat incidents were reported to the NYSDOH's Zoonoses Program and to BCEHFP. In 1999, the Children's Camp Bat Exposure Incident Report form was developed to standardize the reports. Twenty-three different types of incidents could be reported, 13 of which were considered probable rabies exposures requiring consideration of PEP. The form was revised in 2000 to include additional information about the incidents, and in 2001 and 2002 the types of bat incidents reported were limited to the 13 types that require consideration of PEP if the bat is not tested and confirmed negative for rabies. These incidents include: bite; scratch; saliva or nervous tissue contact; direct physical contact with live or dead bat; person touched bat without seeing the part of bat touched; bat flew into person and touched person's bare skin; bat flew into person and touched person's lightweight clothing, and person reports feeling an unpleasant sensation at the point of contact; person with bare feet stepped on bat; person awakens to find a bat in the room; live bat found in room with an unattended infant, child, or person with sensory or mental impairment; person slept in small, closed-in camp cabin, in which bats were swooping past sleeping person; bat found on ground near an unattended infant, child, or person with mental impairment; unidentified flying object hits person and time of day (dusk or dawn), presence of mark where hit, and place where flying object came from (i.e., good site for roosting bats) all support likelihood that it was a bat. The camps reported the bat incidents to the local health department or NYSDOH district offices, which submitted the incident report forms to BCEHFP; that bureau then forwarded the forms to the Zoonoses Program. Staff from the Zoonoses Program and Wadsworth Center taught local and district camp inspectors how to prevent human contact with bats, bat capture techniques, and methods of evacuating a building during an incident.

Reported incidents and additional information from 3 other reporting sources were added to the children's camp database for the final analysis. Information included: 1) specimen history forms for camp-associated bats that were tested at the Rabies Laboratory; 2) the Zoonoses Program rabies exposure and PEP database established by a statewide reporting requirement; and 3) CEH's environmental Health Information and Permitting System (eHIPS).

**Figure 1:**
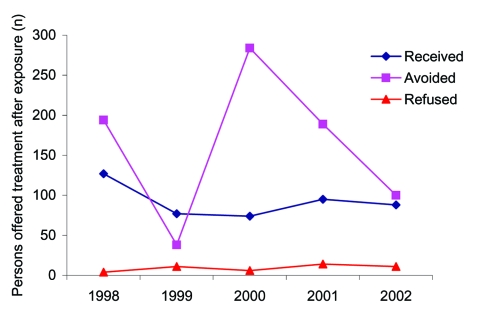
Number of persons who refused, received, or avoided postexposure prophylaxis (PEP) in children's camp bat incidents, New York State, 1998–2002. Treatment status was unknown (not reported to New York State Department of Health) for 117 persons: 9 persons in 1998, 19 persons in 1999, 22 persons in 2000, 33 persons in 2001, and 34 persons in 2002. PEP was avoided because the bats were captured and tested negative for rabies virus.

From 1998 to 2002 during the summer camp season (June through August), 299 bat incidents were reported at 109 of the estimated 2,600 NYS children's camps, involving 1,429 campers and staff (Table). The average and median ages of persons in bat incidents (based on the reported ages of 963 persons) were 14.8 and 13 years, respectively. During the 5-year period, 461 (32.2%) exposed persons (337 campers, 123 staff, 1 unknown status) received PEP (Figure 1). Forty-six persons refused PEP, and treatment status was unknown for 117. Over the 5-year period, bats were submitted for testing, and rabies was ruled out in 52.5% of the incidents. These test results prevented 805 (61.3%) exposed persons (567 campers, 196 staff, 42 unknown status) from having PEP treatment. Of the 209 bats tested from 1998 to 2002, 4 bats collected in 2000 were rabid, and these incidents did not require any treatment for exposure.

**Table:** Children's camp bat incidents and number of persons reported, New York State, 1998-2002*

Bat incidents	1998	1999	2000	2001	2002	Total
Reported incidents (June–August)	45	34	74	74	72	299
No. of incidents with bat submitted for testing (%)	19 (42.2)	5 (14.7)	44 (59.4)	50 (67.5)	43 (59.7)	161 (53.8)
No. of incidents with rabid bat	0	0	4	0	0	4
No. of camps reporting incidents	16	21	46	42	40	109
No. of persons in reported incidents	334	145	386	331	233	1,429

*From 1998 to 2000, all bat incidents at children's camps were requested for reporting. From 2001 to 2002, only bat incidents resulting in concern about potential rabies exposure were requested for reporting.

**Figure 2:**
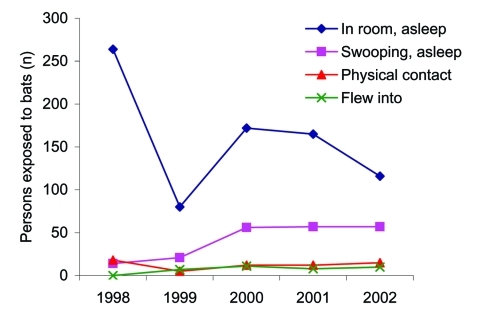
Number of persons exposed to bats by most frequently reported incident types, New York State, 1998–2002. Shown are the 4 most reported exposures of 23 reportable incidents of any type from 1998 to 2000, and of the 13 reportable exposure types from 2001 to 2002. Postexposure prophylaxis was avoided because the bats were captured and tested negative for rabies virus.

Four types of bat exposure reported most frequently accounted for 1,098 (77%) of persons in bat incidents at children's camps (Figure 2). Exposure types were unknown for 69 of the incidents from 1998 to 2002. Specific exposure types (more than 1 type could be reported per incident) and numbers of persons exposed were sleeping where a bat was seen (797), sleeping where bats were swooping (205), direct physical contact with a bat (62), and a bat flying into them (36). The proportion of treatments prevented because of bats testing negative for rabies was 63%, 37%, 26%, and 11%, for these 4 types of exposure, respectively.

## Conclusions

From 1998 to 2002, almost 300 separate bat incidents involving ≈1,500 children and staff at children's camps in New York State were reported. Approximately one third of these persons received PEP because the bats were not captured and tested to rule out rabies. PEP treatment of ≈800 persons was not necessary because the bats were captured and tested negative for rabies.

At an estimated cost of $1,136 per PEP (*2*), this represents healthcare cost savings >$900,000. This estimate underestimates the true cost savings of preventing 5 medical visits during a month for each treated person, transportation costs, coordinating and administering the treatments, opportunity and psychological costs of missing camp, and lost wages.

Most of those involved in bat incidents were campers, which is not unexpected, as most camps have a higher number of campers than staff. Gender often depended on which camp was affected, as many camps are single sex. The 4 most common types of bat exposures requiring PEP are ones in which there is a reasonable probability that rabies exposure has occurred. The 2 most common types of incidents in which PEP was required (sleeping where a bat was seen or was swooping) are preventable by properly bat-proofing camp cabins. PEP can also be avoided with proper bat capture technique and cabin evacuation. In 1 camp, after 5 incidents in a short period, PEP treatment was required in 42 cases. Education on bat-proofing and capture did not prevent 25 subsequent incidents in the same season but did result in bat capture and negative rabies test results in 24 of them, preventing 180 campers and staff members from receiving PEP treatment.

Although only a few human rabies cases are diagnosed each year in the United States, inapparent or unreported bat bites appear to account for most of them (*14*). Equally important, bat exposures strongly affect healthcare costs when rabies cannot be ruled out by capturing and testing bats. Just as it is unacceptable for other wildlife to affect the health and safety of children at camp, keeping bats out of sleeping quarters and other buildings should be part of routine camp safety education, inspection, and certification programs. Although bats are part of the external camp environment, occupied buildings must be bat-proof. If exposures around or in camp buildings do occur, campers and staff must know how to avoid further exposures and how to capture the bat for rabies testing. Systems for reporting camp bat exposures and their consequences will identify this important public health problem and aid public health responses to reduce its impact.
